# A numerical approach to determine the optimal condition of the gas anti-solvent supercritical process for nanoparticles production

**DOI:** 10.1038/s41598-022-15754-x

**Published:** 2022-07-08

**Authors:** Nedasadat Saadati Ardestani, Mitra Amani

**Affiliations:** 1 Department of Nanotechnology and Advanced Materials, Materials and Energy Research Center, Karaj, 14155-4777 Iran; 2 Department of Chemical Engineering, Robat Karim Branch, Islamic Azad University, Robat Karim, 37616-16461 Iran

**Keywords:** Chemical engineering, Chemistry

## Abstract

Supercritical gas antisolvent (GAS) process is an efficient method for nanoparticles production, in which accurate selection of operational condition is essential. Thermodynamic models can be applied for evaluation the phase equilibrium behavior and determination the required precipitation pressure of GAS process. In this research, thermodynamic behavior of (CO_2_—dimethyl sulfoxide (DMSO)) binary system and both of (CO_2_–DMSO-anthraquinone Violet 3RN (AV3RN)) and (CO_2_–DMSO-solvent Yellow 33 (SY33)) ternary systems in the GAS process were studied at different temperatures (308, 318, 328 and 338) K and pressures (1.0–14.0) MPa, using Peng–Robinson equation of state (PR-EoS). The minimum precipitation pressure of AV3RN and SY33 at 308, 318, 328 and 338 K were 7.80, 8.57, 9.78 and 11 MPa and 8, 8.63, 9.5 and 10.77 MPa, respectively. Also, the mole fraction of substances in liquid phase of ternary systems were determined by PR-EoS, at 328 K versus pressure. The accuracy of the obtained results were investigated using the experimental data reported in the literatures.

## Introduction

It has been proved that reducing the size of a substance to the nanometer scale can significantly change its properties^1^. Research on production of various nanoparticles with controlled particle size and particle size distribution have attracted a lot of attention due to their tunable chemical, physical and biological properties with better performance than the original bulk prototype^2,3^.

Pigments and dyes are widely used in various industries for coloration of textiles, foods, plastics, drugs, cosmetics, wax, soap and etc. Particle characteristics of these compounds such as size distribution, morphology and crystal structure can extensively influence on their properties and application fields^4^. Due to wide industrial application of nano pigments and nano dyes, searching possible methods and suitable operational parameters for production of high quality nanoparticles with narrow particle size distribution (PSD) is technically important. However, wide particle size distribution, thermal degradation, high residual solvent in the final product and environmental pollution associated with the use of an organic solvent are the main disadvantages of traditional particle size reduction techniques. Among the alternative methods, supercritical fluid (SCF) processes can be applied for precipitation of different nano-metric materials without or with reduced organic solvent usage. Also, their mass transfer characteristics are helpful for generation of nanoparticles with controlled size and distribution. Special properties of CO_2_ such as nontoxicity, non-flammability, nonpolluting, low price, favorable critical conditions, recyclability, environmentally benign and abundance make it the most commonly used SCF^5^. Based on supercritical CO_2_ (sc-CO_2_) role in the precipitation process as solvent^6,7^, antisolvent^8,9^ and reaction media^10,11^, various types of these processes are designed. Sc-CO_2_ employed as an antisolvent for the materials with very low solubility in it. Various kinds of supercritical antisolvent processes, such as SAS (supercritical antisolvent), GAS (gas antisolvent), PGSS (Particles from gas saturated solutions) and SEDS (supercritical enhanced dispersion of solutions) were applied for precipitation of different pigments; such as Red Lake C and C.I. Pigment Yellow 1^12^, Astaxanthin^13^, quercetin^14^, C.I. Disperse Red 60^15^, Coumarin-7^16^, C.I. Pigment red 177^17^, Bronze Red^18^, C.I. Pigment Green 36^19^ and C.I. pigment blue 15:6^20^.

Despite the importance and wide industrial application of pigments and dyes, a little researches have been performed on production of nanoparticles of these substances. Anthraquinone Violet 3RN (AV3RN) and Solvent Yellow 33 (SY33) are two of widely used them. Molecular structure of these components are shown in Table 1. AV3RN is an organometallic pigment and one of the synthetic anionic dyes, especially used in textile industry. SY33 (Quinoline Yellow) is an anionic dye applied as a colorant in textile, plastic, medicine, cosmetic, rubber and many other industries.

**Table 1:** Critical and physical properties of pure solvents and solutes.

Component	T_m_^a^ (K)	T_b_^b^ (K)	T_c_^c^ (K)	P_c_^d^ (bar)	$\omega$ ^e^	Z_c_^f^	V_c_^g^ (cm^3^ mol^−1^)	H_f_^h^ (kj mol^−1^)	H_v_^i^ (kj mol^−1^)	H_fus_^j^ (kj mol^−1^)
Anthraquinone Violet 3RN (AV3RN)	534.64	867.87	1054.46	16.90	1.5	0.12	1155.87	203.90	204.66	46.59
Solvent yellow 33 (SY33)	496	707.95	902	28.06	1.3	0.19	729.3	133.10	113.91	34.88
CO_2_	–	–	304.18	73.8	0.225	–	–	–	–	–
DMSO	–	462	706.9	58.5	0.45	–	–	–	–	–
Anthraquinone Violet 3RN (AV3RN)						Solvent yellow 33 (SY33)				
	

^a^Normal melting point.

^b^Normal boiling point.

^c^Critical temperature.

^d^Critical pressure.

^e^Acentric factor.

^f^Critical compressability factor.

^g^Critical volume.

^h^Standard enthalpy of formation at 298 K.

^i^Standard enthalpy of vaporazation at 298 K.

^j^Standard enthalpy of fusion.

Generally, textile dyeing is known as a process with remarkable water consumption and also it is the second largest polluter of water globally^21^. Alternatively, utilization of sc-CO_2_ as dyeing medium, instead of water, provide great opportunities for significant water savings along with reducing the amount of discharged wastewater to the environment leading to decrement the environmental pollution. Due to necessity of dissolving dyes in sc-CO_2_ phase for penetration to textile fibers, supercritical dyeing (SCD) process efficiency is significantly depends on dye solubility in sc-CO_2_. It has been confirmed in several researches that production of nanoparticles with high surface area is the most efficient approach for solubility enhancement of different components^7–9,22,23^. Solubility of AV3RN in sc-CO_2_ with/without methanol as co-solvent has been measured in temperatures range of 308–348 K and pressures range of 100–350 bar in our previous work^24^. It was shown that AV3RN solubility in the binary (Sc-CO_2_ + AV3RN) and ternary (Sc-CO_2_ + AV3RN + methanol) systems were in the range of 4.7 × 10^−7^ to 5.5 × 10^−6^ and 4.4 × 10^−6^ to 5.8 × 10^−5^ mol fraction, respectively. Solubility of SY33 in sc-CO_2_ has not yet been reported, but according to available data for solubility of some other similar structure dyes^25^, it can be concluded that solubility of SY33 in sc-CO_2_ is also poor. Based on poor solubility of both substances in sc-CO_2_, GAS anti solvent process can be one of the best techniques for production of AV3RN and SY33 nanoparticles.

In this semi batch process, first the solute is completely dissolved in an organic solvent, then sc-CO_2_ is injected into this stationary liquid phase with special flow rate. Sc-CO_2_ dissolution into organic solvent leads to a large volume expansion and reduction of liquid density. This is associated with a decrement in solvation power of organic solvent and a sharp increment of supersaturation degree within the liquid phase. Degree of supersaturation which defines as the ratio of solute concentration to its equilibrium solubility, is the driving force of precipitation in GAS process, impressing the precipitates nucleation and growth rates^26^. Therefore, severe reduction of solid solute solubility causes to precipitation of pure solute nanoparticles which can be separated from the expanded liquid phase by high pressure filtration. More purity, narrow particle size distribution and easier quality control in terms of size, morphology and crystallinity of deposited nanoparticles along with lower operational temperature and negligible levels of particles contamination to organic solvent are the most important preferences of GAS process^12,27^. Due to direct effect of operational condition on equilibrium solubility and supersaturation of solution and consequently on quality of deposited particles, it can be concluded that nanoparticles with desired characteristics are not produced at arbitrary conditions and selection the appropriate operational conditions is so important.

Generally, thermodynamic modelling can be helpful in reduction of required time and energy of experimental studies. Simultaneous presence of solid solute, organic liquid solvent and sc-CO_2_ anti solvent with a certain solubility of solute and sc-CO_2_ in the solvent is one of the thermodynamic aspects of the GAS process. Additionally, phase equilibrium of antisolvent-solvent mixture has determinative role in controlling the particles deposition in this process. So, the optimal conditions of nanoparticles formation in the GAS process should be determined by volume expansion and thermodynamic modeling. Also, thermodynamic modelling can be helpful for detailed understanding the phase behavior of the ternary system (gas/liquid/ solid), analyzing the effect of operational conditions and finally evaluation the feasibility and optimization of GAS process. Up to now, different thermodynamic models using an equation of state (EoS) for calculation the fugacity of three phases, have been applied for determination the solute and sc-CO_2_ solubilities in liquid solvent and calculation the volume expansion of liquid phase. Peng-Robinson (PR) with conventional quadratic mixing rules (vdW2) is one of the most popular EoS used for this purpose^28,29^.

In current work, for the first time, the optimal operational conditions of AV3RN and SY33 nanoparticles precipitation via GAS process specified through modeling the volume expansion and phase equilibrium via PR-EoS with vdW2 mixing role. In this system, AV3RN and SY33, Dimethyl sulfoxide (DMSO) and sc-CO_2_ were considered as solutes, solvent and antisolvent, respectively. DMSO is one of the commonly used solvent in supercritical antisolvent processes, due to its high miscibility with sc-CO_2_^30–32^. Also, remarkable high volumetric expansion of DMSO with sc-CO_2_ is reported in several researches^29,33^.

## Theoretical studies

### Thermodynamic framework

For feasiblity analysis of AV3RN and SY33 nanoparticles production via GAS process, determination the optimum process condition and understanding the solute precipitation mechanism are performed by thermodynamic modeling. In this research, the Peng-Robinson equation of state (PR-EoS) was utilized for investigation the phase equilibrium and volume expansion of the liquid phase.

#### Phase equilibrium and volume expansion analysis

In this study, sc-CO_2_ as antisolvent (1) in gas phase (g), DMSO as solvent (2) in liquid phase (l) and AV3RN and SY33 as solutes (3) in solid phase (s) are in equilibrium with each other. The equilibrium concentration of each of these components is an effective thermodynamic parameter which determines the maximum accessible supersaturation in the system. Equality of pressure, temperature and fugacity of these components in all the phases are the equilibrium criteria of these phases. Also, due to mixing of the liquid and gas phases, the mass transfer resistance between these two phases can be ignored^30^. Thus, the equilibrium condition can be explained by the following equilibrium equations:

1 $$ y_{1} \,\phi_{1}^{v} \,P\, = \,x_{1} \,\phi_{1}^{l} \,P $$

2 $$ y_{2} \,\phi_{2}^{v} \,P = \,x_{2} \,\phi_{2}^{l} \,P $$

3 $$ y_{3} \,\phi_{3}^{v} \,P\, = \,x_{3} \,\phi_{3}^{l} \,P $$

4 $$ \,\phi_{3}^{s} \,P\,\, = \,x_{3} \,\phi_{3}^{l} \,P $$

where $\phi_{i}^{{}}$, *y*_*i*_ and *x*_*i*_ are the fugacity coefficient and equilibrium mole fraction of component “i” in gas and liquid phases, respectively. In Eq. (4), it was assumed that the solid phase is pure and the solubility of the solvent and the antisolvent in this phase is insignificant. Obviously, the sum of components mole fractions in both of the liquid (*x*_*i*_) and gas (*y*_*i*_) phases are equal to one^34^. Accordingly, a system of six equations with six unknowns of molar compositions in liquid and gas phases is obtained.

The following relationship along with an appropriate equation of state can be used for calculation the fugacity coefficient of each component in the liquid and gas phases:

5 $$ RT\ln \phi_{i} = \, - RT\ln Z\, + \,\int_{V}^{\infty } {\left[ {(\frac{\partial P}{{\partial n_{i} }})_{{T,V,n_{j \ne } n_{i} }} - \frac{RT}{V}} \right]} \,dV $$

Peng- Robinson equation of state (PR-EoS) have been used as follows:

6 $$ P = \frac{RT}{{\nu - b}} - \frac{a(T)}{{\nu \,\,(\nu + b) + b\,(\nu - b)}} $$

where *P*, *T* and *R* are the absolute pressure (MPa), temperature (K) and the universal gas constant (8.314 J mol^−1^ K^−1^), respectively. Also, *ν* shows the solute molar volume (m^3^ mol^-1^) which was computed by Fedors method^35^. Moreover, *a(T)* and *b* terms account for interactions between the species in the mixture and excluded volume of the mixture species, respectively. Generally, these two parameters are obtained via quadratic mixing rules in terms of the equilibrium compositions. The van der Waals mixing rules for *a(T)* and *b* is given as:

7 $$ a(T)\, = \,\sum\limits_{i} {\sum\limits_{j} {x_{i} } } x_{j} a_{ij} (T) $$

8 $$ b\, = \,\sum\limits_{i} {\sum\limits_{j} {x_{i} } } x_{j} b_{ij} $$

where *x* is the mole fraction. Also, *a*_*ij*_*(T)*, the cross energetic parameter, and *b*_*ij*_ can be calculated as follows:

9 $$ a_{ij} (T) = \,\sqrt {a_{i} (T)\,a_{j} (T)} \,(1 - k_{ij} ) $$

10 $$ b_{ij} \, = \,\frac{{(b_{i} + \,b_{j} )}}{2}(1 - l_{ij} ) $$

where *k*_*ij*_ and *l*_*i*j_ are the binary interaction parameters; *a(T)* and *b* parameters are dependent on the critical and physical properties of the components:

11 $$ a(T) = \frac{{0.45724R^{2} T_{c}^{2} }}{{P_{c} }}\, \times \alpha (T_{r} ,\,\omega ) $$

12 $$ b = \frac{{0.07780RT_{c} }}{{P_{c} }} $$

where

13 $$ \alpha (T_{r} ,\,\omega ) = [1 + m(1 - T_{r}^{0.5} )]^{2} \quad \& \quad m = 0.37464 + 1.5422\omega - 0.26992\omega^{2} $$

where *ω* is acentric factor and *T*_*r*_ is the reduced temperature (T_r_ = T/T_c_). However, the PR-EoS is inappropriate for modeling the phase behavior of solid phase. So, modified form of the proposed equation by de la Fuente Badilla et al.^36^ is applied for calculation of solid fugacity coefficient ($\,\varphi_{3}^{s}$):

14 $$ \ln \phi_{3}^{s} \, = \,\ln \phi_{3}^{l,pure} + \frac{{\Delta H_{tp} }}{R}(\frac{1}{{T_{tp} }} - \frac{1}{T}) + \frac{{v_{tp} }}{RT}(P - P_{tp} ) $$

This equation relates the fugacity coefficient of the solid solute ($\varphi_{3}^{s}$) to the fugacity of the sub-cooled liquid ($\varphi_{3}^{l}$) at temperature *T* and pressure *P*, which can be calculated by the PR-EOS. For calculation of $\varphi_{3}^{s}$, the heat of fusion at the triple point ($\Delta H_{tp}$), the triple point temperature (*T*_*tp*_), the triple point pressure (*P*_*tp*_) and the molar volume of the solute at the triple point (*v*_*tp*_) should be specified. These characteristics for AV3RN and SY33 components are listed in Table 2.

**Table 2:** Thermophysical properties of anthraquinone violet 3RN and solvent yellow 33 (solid solutes), required in Eq. (14).

Substance	MW^a^ (kg kmol^-1^)	T_tp_^b^ (K)	P_tp_^c^ (Pa)	v_tp_^d^ (cm^3^ mol^-1^)	ΔH_tp_^e^ (kj mol^-1^)
Anthraquinone Violet 3RN (AV3RN)	578.6	534.64	4.79	273.01	251.25
Solvent yellow 33 (SY33)	273.3	513.00	283.64	195.09	148.79

^a^Molecular weight.

^b^Triple point temperature.

^c^Triple point pressure.

^d^Triple point volume.

^e^Heat of fusion at the triple point.

The volume expansion of liquid phase has a decisive role in GAS process and computation of this parameter is essential for determination of optimum process condition. The definition of the relative molar volume change is given as follows^30,36^:

15 $$ \frac{\Delta v}{v} = \,\frac{{v(T,P) - \,v_{0} (T,P_{0} )}}{{v_{0} (T,P_{0} )}} $$

In this relation, *v(T, P)* is the molar volume of the liquid solution at the system temperature and pressure and *v*_*0*_*(T, P*_*0*_*)* stands for the molar volume of the pure solvent at the system temperature and reference pressure (P_0_) (usually atmospheric pressure). If CO_2_ dissolution accompanied with reducing the molar volume, the sign of this parameter became negative and vice versa.

#### Optimization algorithm: particle swarm optimization (PSO)

Process optimization is an important part of a process design where input process parameters are optimized to reach satisfactory output parameters. Numerous optimization techniques have been developed for different optimization problems. Particle Swarm Optimization, commonly referred as PSO, is one of the most commonly utilized optimization algorithms for solving continuous nonlinear optimization problems, firstly introduced by Kennedy and Eberhart^37^. Generally, it is a computational method that optimizes an objective function of mathematical problem by iteratively trying to improve a candidate solution with regard to a given measure of quality^38^. PSO is a popular technique with very easy execution only needs a few programming code lines. Also, simplicity of required mathematical operators makes it computationally economical in terms of both memory requirements and speed. PSO algorithm is created by natural swarms that are formed of volume-less particles with random velocities, each of which shows a feasible solution. This algorithm finds the optimum solution by moving the particles in the solution space^39^. PSO performs searching via a swarm of particles that updates from iteration to iteration. To find the optimum solution, each particle moves in the direction to its previously best position and the global best position in the swarm^40^.

### Determination the physicochemical properties of AV3RN and SY33

Physicochemical properties of CO_2_, organic solvent (DMSO) and solid solutes (AV3RN and SY33) are needed parameters in modelling the supercritical processes by cubic equation of states. Required characteristics of CO_2_ and DMSO including critical properties, boiling and melting point, acentric factor and molar volume are reported in literatures. However, these properties for complex molecules such as pigments and dyes are not available and their experimental determination is so difficult. In these situations, suitable group contribution methods can be applied for computation of required solids properties.

In this work, critical pressure (*P*_*c*_), critical temperature (*T*_*c*_) and normal boiling point (*T*_*b*_) are estimated by Marrero and Gani method^41^. The acentric factor (*ω*) and solid molar volume (ν) are calculated by Constantinou—Gani^42^ and Fedors^35^ methods, respectively. Also, the heat of fusion at the triple point ($\Delta H_{tp}$) and the triple point temperature (*T*_*tp*_), which can be well estimated to the melting point^26^, of AV3RN and SY33 molecules are obtained from differential scanning calorimetry (DSC) analysis. The critical and physicochemical properties of AV3RN and SY33, DMSO, and CO_2_ are presented in Table 1. Also, required physical characteristics of Eq. (14) for AV3RN and SY33 molecules are presented in Table 2.

## Result and discussion

Analyzing the phase behavior through thermodynamic modeling is a suitable approach for assessing the optimal conditions of nanoparticles production via GAS process. In current work, the fluid phase behavior in binary (CO_2_–DMSO) and ternary (CO_2_–DMSO-AV3RN) and (CO_2_–DMSO- SY33) systems was investigated using PR-EoS.

### Binary system (CO_2_–DMSO)

**Figure 1:**
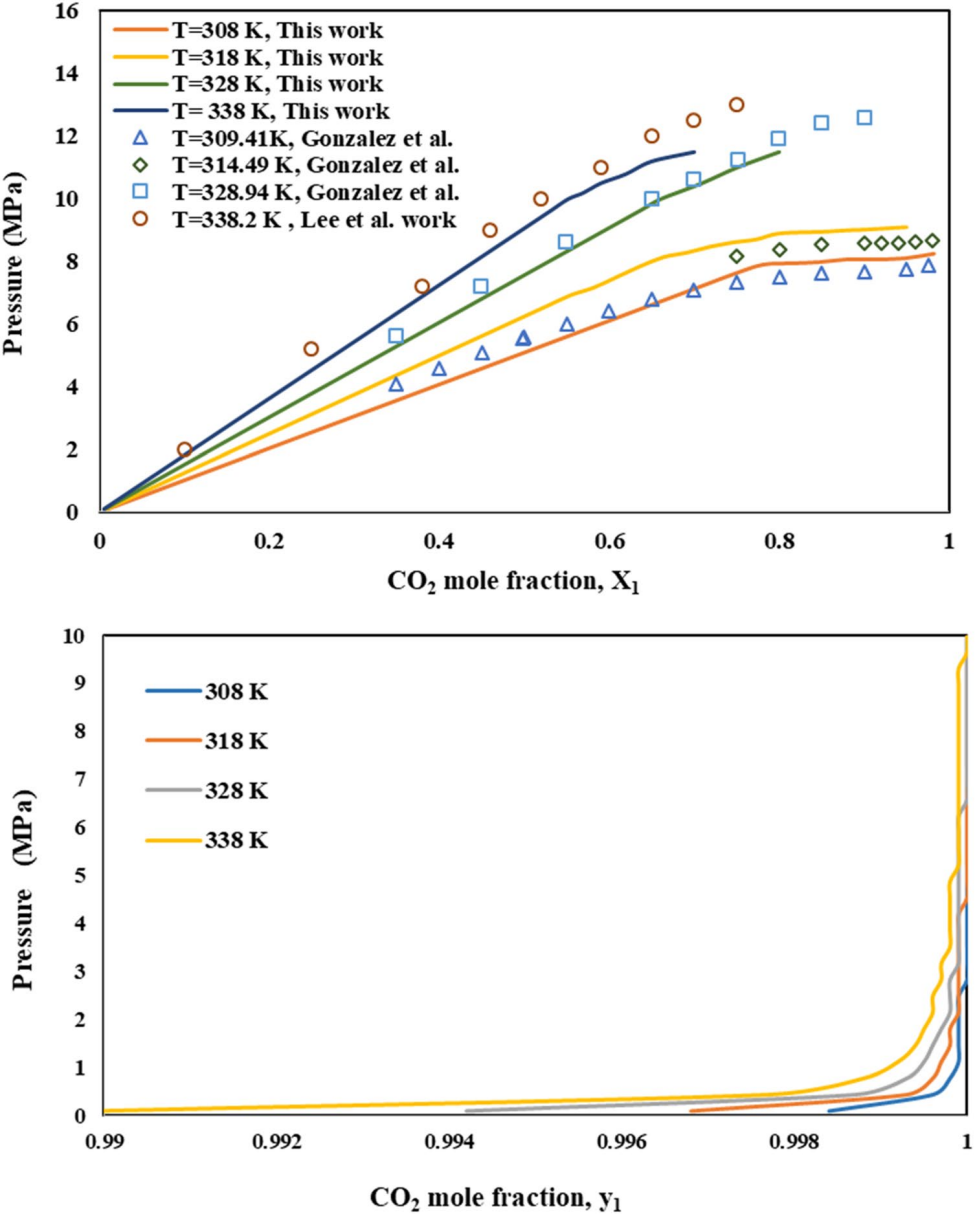
Comparison of VLE of DMSO–CO_2_ system calculated according to PR at 308, 318, 328 and 338 K.

Degree of CO_2_ solubility in organic solvent, as a function of temperature and pressure, is a key parameter in fine particles precipitation by the GAS process. The vapor-liquid equilibrium (VLE) behavior of CO_2_ – DMSO binary system as a function of temperature and pressure is shown in Fig. 1. For validation the accuracy of the considered thermodynamic model, calculated equilibrium mole fraction of dissolved sc-CO_2_ in DMSO (x_1_) is compared with the experimental data reported by Gonzalez et al*.*^43^ and Lee et al*.*^17^, in Fig. 1a. As is evident, model results are well consistent with the reported experimental data.

As expected, CO_2_ solubility in organic solvent (x_1_) increases by pressure increment and temperature decrement (Fig. 1). Bakhshi et al*.*^44^ have presented similar results with calculation the solubility of CO_2_ in toluene and ethanol with SRK-vdW2 and PR-HV models, respectively. Also, the same trend was reported by Yao et al*.*^45^ for CO_2_–acetone system and Ghoreishi et al*.*^30^ for CO_2_–DMSO system, in which the VLE behavior of both systems is calculated by PR-EoS.

**Figure 2:**
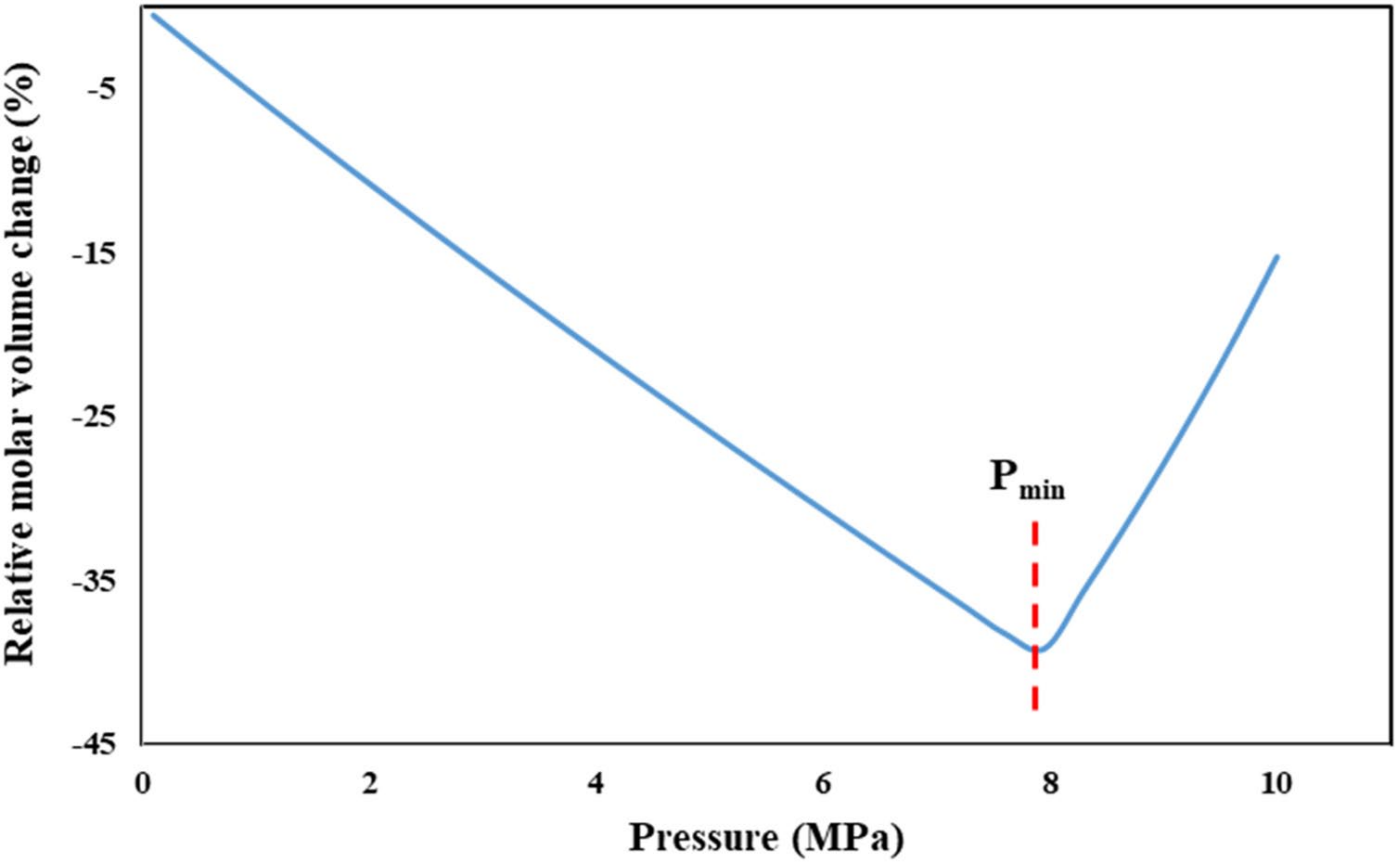
Relative molar volume change of the liquid phase vs. pressure, for the binary system (CO_2_–DMSO) at 328 K.

**Figure 3:**
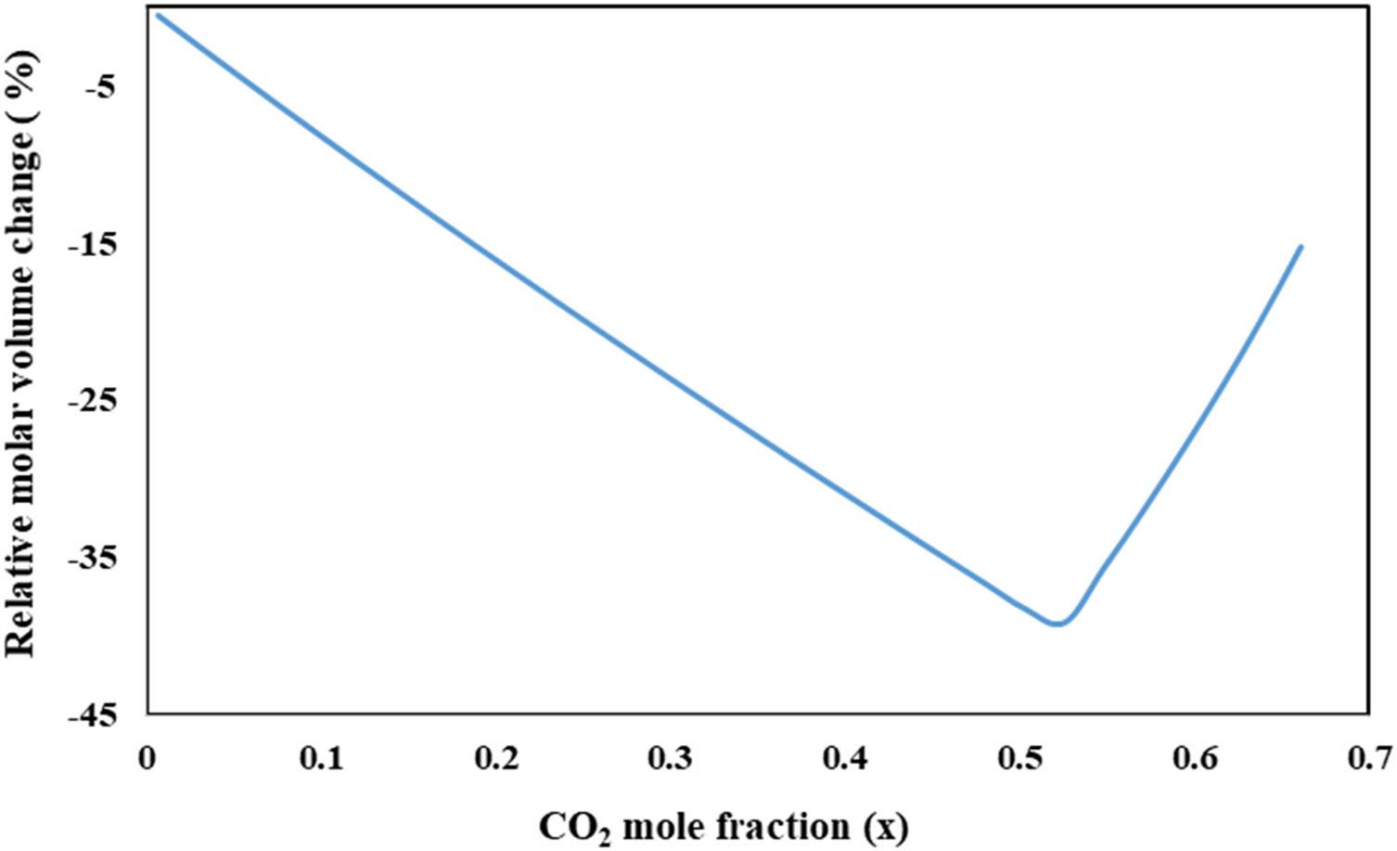
Relative molar volume change of the liquid phase vs. CO_2_ mole fraction in liquid phase, for the binary system (DMSO–CO_2_) at 328 K.

For determination the optimum operating pressure, the relative molar volume change is plotted as a function of pressure at the different temperatures. The optimum operating pressure of the GAS process has to be selected in such a way that the relative molar volume change of the CO_2_- solvent mixture shows a minimum value. So, a minimum point of pressure (P_min_) along with a sharp increment of volume expansion in this plot is an accepted criterion for this purpose. Based on reported results in de la Fuente et al*.* research^36^, the P_min_ value indicates the minimum required pressure in GAS process for precipitation of about 95% of the solute in the form of fine particles. Thus, the operating pressure should be above the pressure of this minimum point (P_min_). Variation of relative molar volume for CO_2_-DMSO binary system as a function of pressure at constant temperature (328 K) is presented in Fig. 2. P_min_ value of 7.95 MPa is evident in this graph. As shown in Fig. 3, trend of relative molar volume change of this binary system versus the dissolved CO_2_ mole fraction in liquid phase at 328 K is completely similar to Fig. 2. Molar volume contraction was observed by increasing dissolved CO_2_ concentration in DMSO (mole fraction) up to a specified value (x_1min_ = 0.52). Afterwards, further CO_2_ dissolution in DMSO change the molar volume status to molar volume expansion.

Observed trend can be explained based on definition of liquid molar volume of solution (ν)^46^:

16 $$ \nu = x_{1} \,\overline{\nu }_{1} \, + \,x_{2} \,\overline{\nu }_{2} $$

where $\overline{\nu }_{1} \,$ and $\overline{\nu }_{2} \,$ are partial molar volume of component 1 (CO_2_) and 2 (DMSO) in a binary mixture of them. Regardless of whether ν_2_ is bigger or smaller than ν_1_, generally, the molar volume of the solution (ν) is frequently lower than that of the pure solvent (ν_2_), except for cases with very high dissolution of component 1 (high value of x_1_) in the organic solvent. The relation between $\overline{\nu }_{1} \,$ and $\overline{\nu }_{2} \,$ can be defined as follow:

17 $$ \overline{\nu }_{1} \, - \,\overline{\nu }_{2} \, = \,(\frac{\partial \nu }{{\partial x_{1} }})_{P,T} $$

Given that liquid molar volume of solution is a function of pressure, temperature and CO_2_ mole fraction, $\frac{d\nu }{{dx_{1} }}$ can be defined as follows:

18 $$ \frac{d\nu }{{dx_{1} }} = \,(\frac{\partial \nu }{{\partial P}})_{{T,x_{1} }} \frac{dP}{{dx_{1} }} + \,(\frac{\partial \nu }{{\partial x_{1} }})_{P,T} $$

In this equation, ($(\frac{\partial \nu }{{\partial P}})_{{T,x_{1} }} \frac{dP}{{dx_{1} }}$)) is a negative and small value, whereas $\,(\frac{\partial \nu }{{\partial x_{1} }})_{P,T}$ is negative at the first and becomes positive with increasing CO_2_ dissolution in organic solvent. Accordingly, with initiation of CO_2_ dissolution in DMSO, the liquid molar volume of solution reduces at the first (as $\overline{\nu }_{1} \, \prec \,\overline{\nu }_{2} \,$). After that, by increasing dissolved CO_2_ concentration in DMSO, $\overline{\nu }_{1} \,$ becomes equal with $\overline{\nu }_{2} \,$ and the liquid molar volume of solution reaches to its lowest value (the minimum point of corresponding graph). Subsequently, further CO_2_ dissolution leading $\overline{\nu }_{1} \,$ becomes larger than $\overline{\nu }_{2} \,$ and volume expansion be revealed. These trends are confirmed by other researchers, too. Chen et al*.*^31^ applied Volume-Translated Peng-Robinson (VTPR) EoS for calculation the relative molar volume change of liquid phase in binary systems of CO_2_ and different solvents including acetone, DMSO, ethanol, and ethyl acetate. Different P_min_ values obtained at 308 K as 5.40, 7.65, 7.10, and 6.15 MPa K for acetone, DMSO, ethanol, and ethyl acetate, respectively. These values clearly show the effect of solvent type on solute precipitation in GAS process. Ghoreishi et al*.*^30^ have also reported the P_min_ values of 7, 7.74 and 8.5 MPa for CO_2_-DMSO binary system at different temperatures (308, 313 and 319 K). They calculated the relative molar volume variation vs. pressure using PR-EoS with linear combination of Vidal and Michelsen mixing rules (PR-LCVM). Reported results in Mamata Mukhopadhyay research^28^ show that partial molar volume of DMSO and CO_2_ are descending and ascending functions of dissolved CO_2_ mole fraction, respectively.

**Figure 4:**
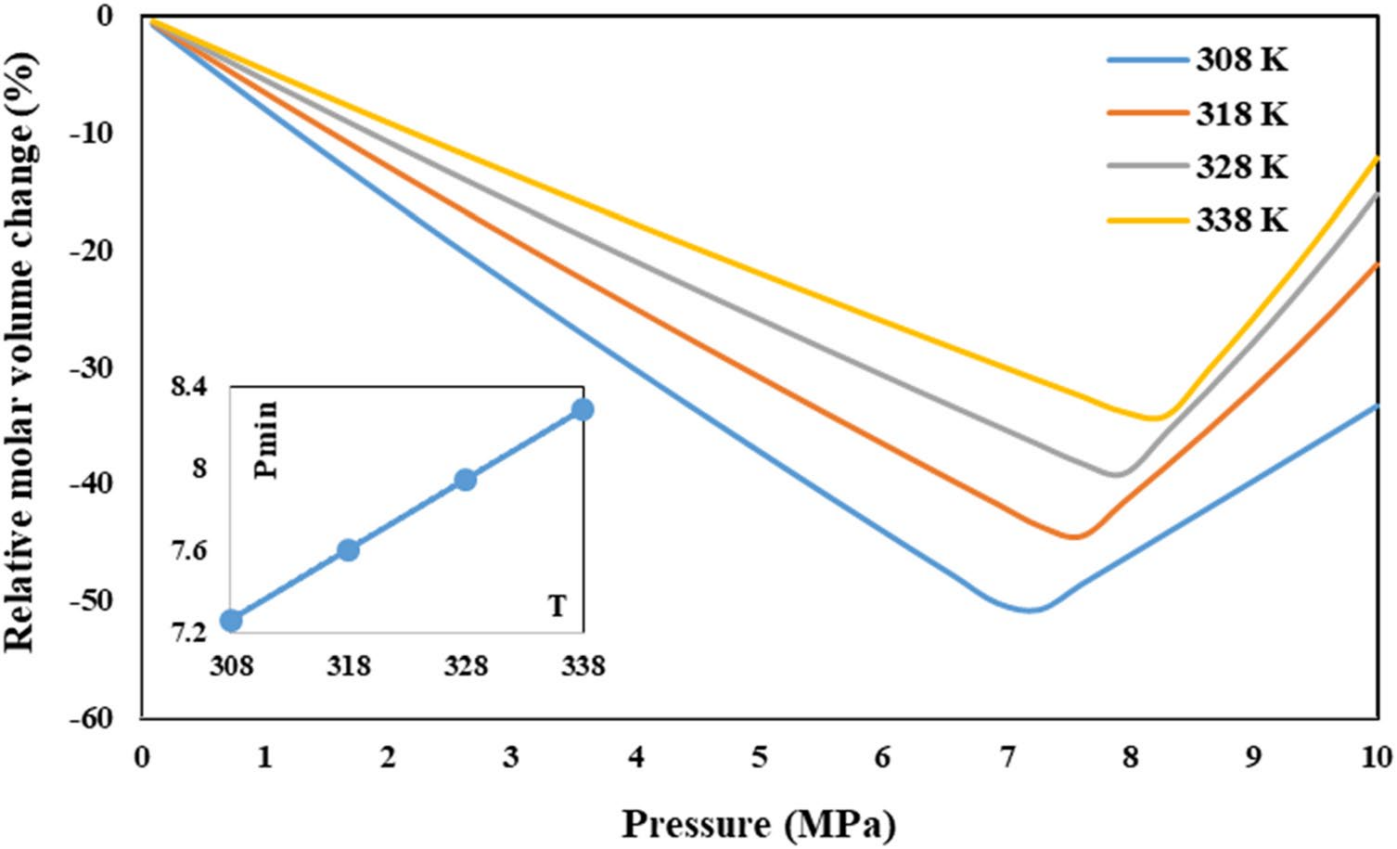
Relative molar volume change of the liquid phase vs. pressure, for the binary system (CO_2_–DMSO) at various temperatures (308, 318, 328 and 338 K).

Variations of relative molar volume against pressure for CO_2_-DMSO binary system at various temperatures (308, 318, 328 and 338 K) were shown in Fig. 4. Accordingly, the computed *P*_min_ values were 7.27, 7.61, 7.95 and 8.29 MPa at 308, 318, 328 and 338 K, respectively. As is evident, required P_min_ for solute precipitation is increased with temperature increment which is related to reducing the solubility of sc-CO_2_ gas in DMSO due to temperature increment.

The binary interaction parameters of Eqs. 9 and 10 (*k*_*ij*_ and *l*_*ij*_) have been optimized by PSO algorithm for each temperature and optimally fitted parameters are shown in Table 3.

**Table 3:** Binary interaction parameters from PR-EoS with the vdW2 mixing rules for the binary system (CO_2_ (1)—DMSO (2)).

System	CO_2_-DMSO		
Temperature (K)	${\mathrm{k}}_{12}$	${\mathrm{l}}_{12}$	P_min_ (MPa)
308	0.030	0.034	7.27
318	0.023	0.059	7.61
328	0.024	0.079	7.95
338	0.089	0.094	8.29

### Ternary systems of (CO_2_–DMSO-AV3RN) and (CO_2_–DMSO-SY33)

**Figure 5:**
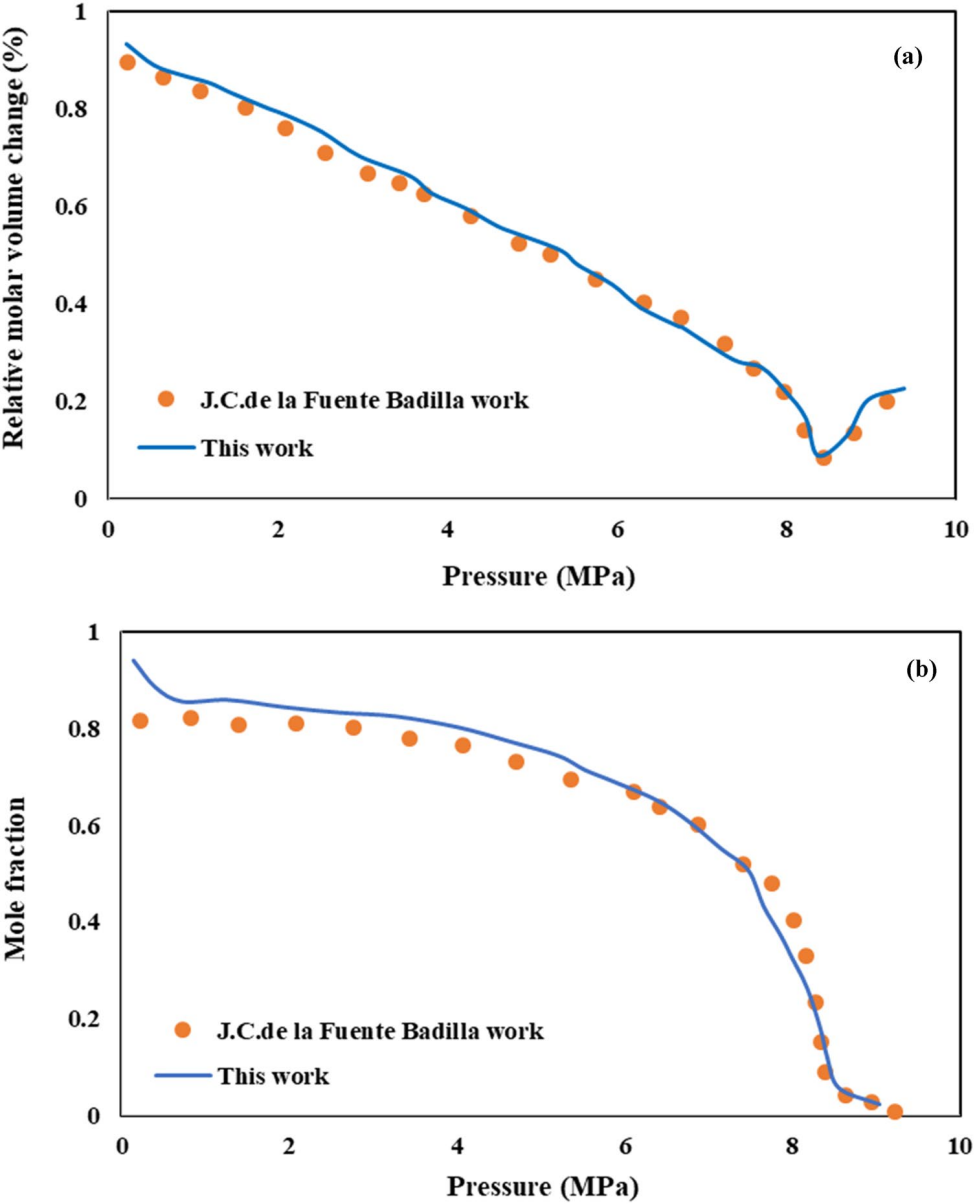
Comparison the results of model and experimental data for CO_2_**–**toluene–naphthalene ternary system **(a)** relative change in molar volume, **(b)** variation of solute (naphthalene) mole fraction.

For determination the optimum operational conditions of AV3RN (pigment) and SY33 (dye) nanoparticles precipitation via GAS process, the phase behavior of (CO_2_–DMSO-AV3RN) and (CO_2_–DMSO-SY33) ternary systems was investigated by PR-EoS with vdW2 mixing rule. For validation the model results in a ternary system, relative molar volume change and variation of solute (naphthalene) mole fraction in CO_2_**–**toluene–naphthalene ternary system were determined by this model and compared with experimental data reported by Fuente Badilla^36^. As shown in Fig. 5a,b, good consistence between model results and experimental data confirms the model accuracy.

**Figure 6:**
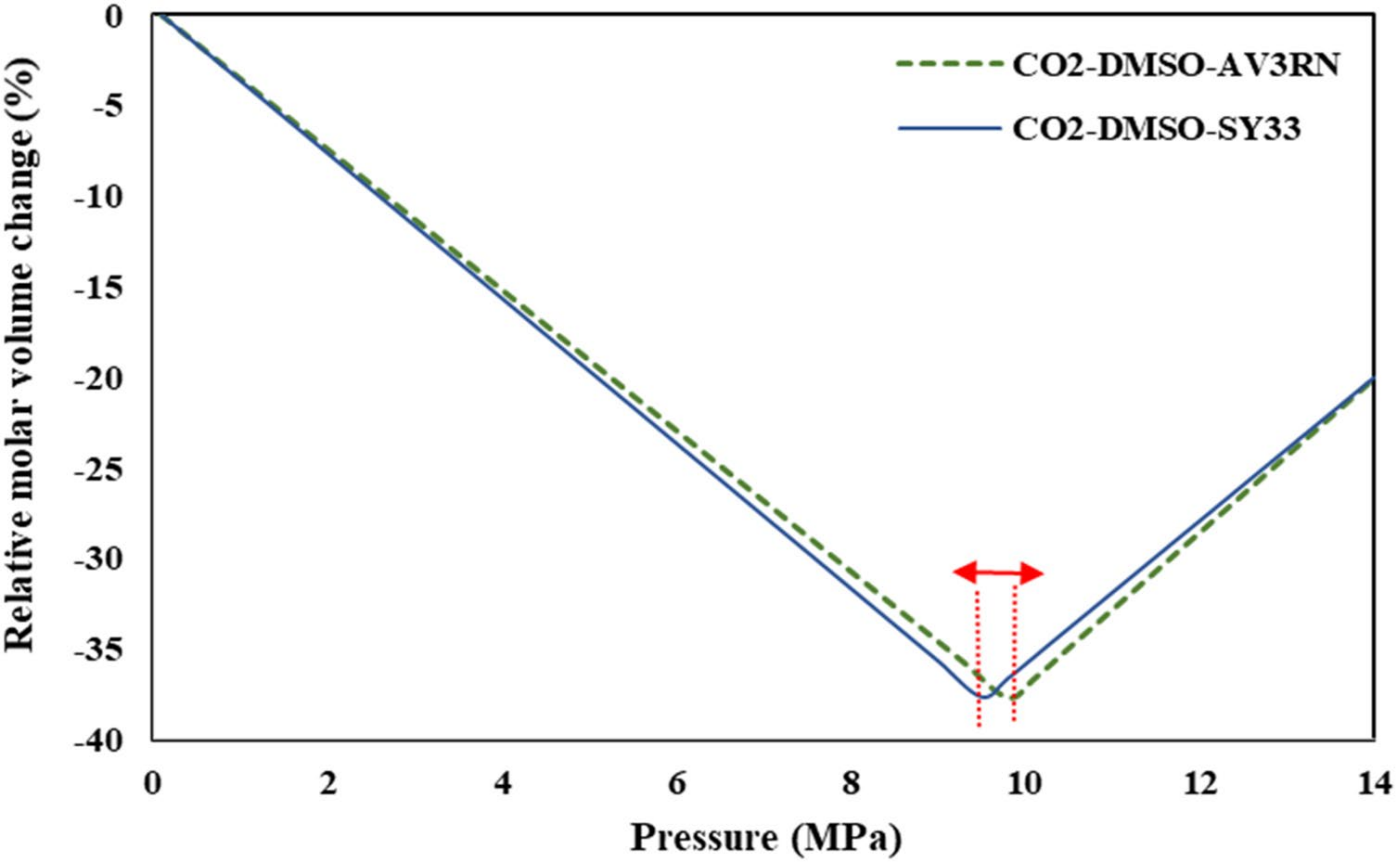
Relative molar volume change of the liquid phase vs. pressure, for the ternary systems (CO_2_–DMSO– AV3RN) and (CO_2_–DMSO– SY33) at 328 K.

**Figure 7:**
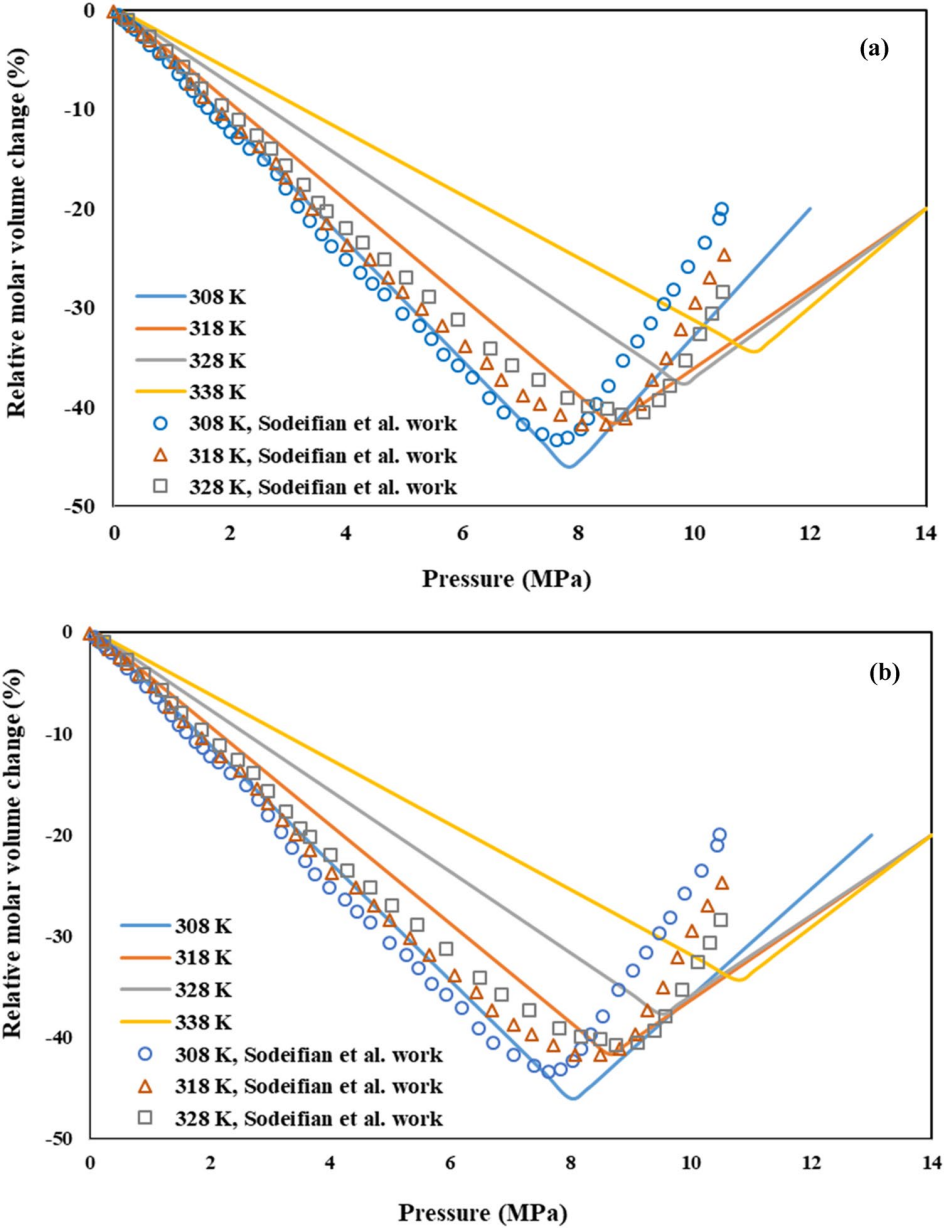
Relative molar volume change of the liquid phase vs. pressure, for the ternary systems **(a)** CO_2_–DMSO–AV3RN and **(b)** CO_2_–DMSO–SY33, at various temperatures (308, 318, 328 and 338 K).

The binary interaction parameters of the (CO_2_–DMSO-AV3RN) and (CO_2_–DMSO-SY33) ternary systems, based on PR-EoS, are reported in Tables 4 and 5, respectively. The relative molar volume change of both of these ternary systems at 328 K was shown in Fig. 6. As can be seen, the lowest relative molar volume change of the liquid phase is appeared at 9.8 and 9.5 MPa for AV3RN and SY33, respectively. So, AV3RN and SY33 nanoparticles can optimally precipitated above the relevant P_min_ values and at the same temperature, SY33 precipitates a bit sooner. The behavior of the relative molar volume change versus pressure at different temperatures (308, 318, 328 and 338 K) is shown in Fig. 7a and b for CO_2_-DMSO-AV3RN and CO_2_-DMSO-SY33 ternary systems, respectively. Also, the obtained results were validated with the experimental data of CO_2_-DMSO-phthalocyanine green (Pc-G) ternary system, reported by Sodeifian et al*.*^47^. As is evident, model results and the experimental data are in good agreement with each other, which approving the model precision. As previously described, increasing temperature leads to higher minimum pressure (P_min_) value. The obtained P_min_ values at 308, 318, 328 and 338 K were 7.80, 8.57, 9.78 and 11 MPa and 8.0, 8.63, 9.50 and 10.77 MPa for AV3RN and SY33, respectively. Comparision the Figs. 7 and 3 indicates that the calculated P_min_ values for both of ternary systems (Fig. 7) were higher than that of the CO_2_-DMSO binary system (Fig. 3) at corresponding temperatures. Difference between P_min_ values of binary and ternary systems highlights the significance of selection the optimum operating conditions for each individual ternary system. At pressures higher than P_min_, a high supersaturation status of solute is expected for successful precipitation of fine solid particles. So, the operation pressure should be selected above the P_min_ value, which was confirmed by others, too^31,48,49^.

**Table 4:** Binary interaction parameters from PR-EoS with the vdW2 mixing rule for the ternary system (CO_2_ (1)—DMSO (2)—AV3RN (3)).

System	CO_2_-DMSO-AV3RN						
Temperature (K)	${\mathrm{k}}_{12}$	${\mathrm{k}}_{13}$	${\mathrm{k}}_{23}$	${\mathrm{l}}_{12}$	${\mathrm{l}}_{13}$	${\mathrm{l}}_{23}$	P _min_ (MPa)
308	0.015	0.019	0.012	0.0025	0.025	0.023	7.8
318	0.015	0.200	− 0.013	− 0.0026	0.025	0.258	8.6
328	0.056	0.025	− 0.018	− 0.012	0.019	0.339	9.8
338	0.012	0.020	− 0.015	− 0.0027	0.005	0.458	11

**Table 5:** Binary interaction parameters from PR-EoS with the vdW2 mixing rule for the ternary system (CO_2_ (1)—DMSO (2)–SY33 (3)).

System	CO_2_-DMSO-SY33						
Temperature (K)	${\mathrm{k}}_{12}$	${\mathrm{k}}_{13}$	${\mathrm{k}}_{23}$	${\mathrm{l}}_{12}$	${\mathrm{l}}_{13}$	${\mathrm{l}}_{23}$	P_min_ (MPa)
308	0.015	0.009	0.002	0.001	−0.005	−0.310	7.99
318	0.015	0.008	0.006	0.001	−0.006	0.310	8.63
328	0.015	0.007	0.005	0.002	−0.007	−0.21	9.50
338	0.015	0.006	0.002	0.006	−0.005	−0.10	10.76

**Figure 8:**
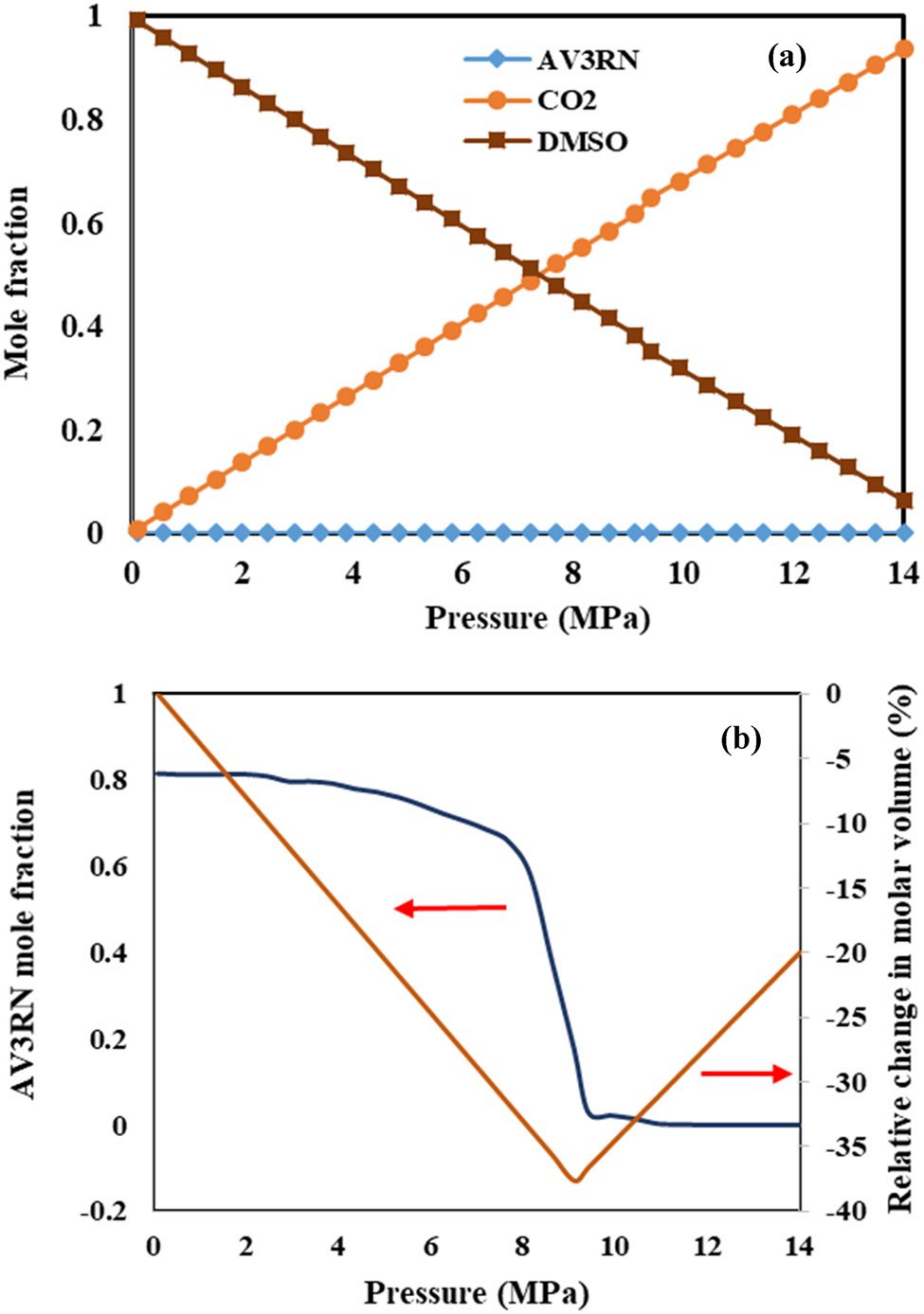
**(a)** Mole fraction of components in the liquid phase of the ternary system of CO_2_–DMSO–AV3RN at 328 K, calculated by the PR- EoS, and **(b)** Mole fraction of AV3RN along with relative change in molar volume at 328 K.

**Figure 9:**
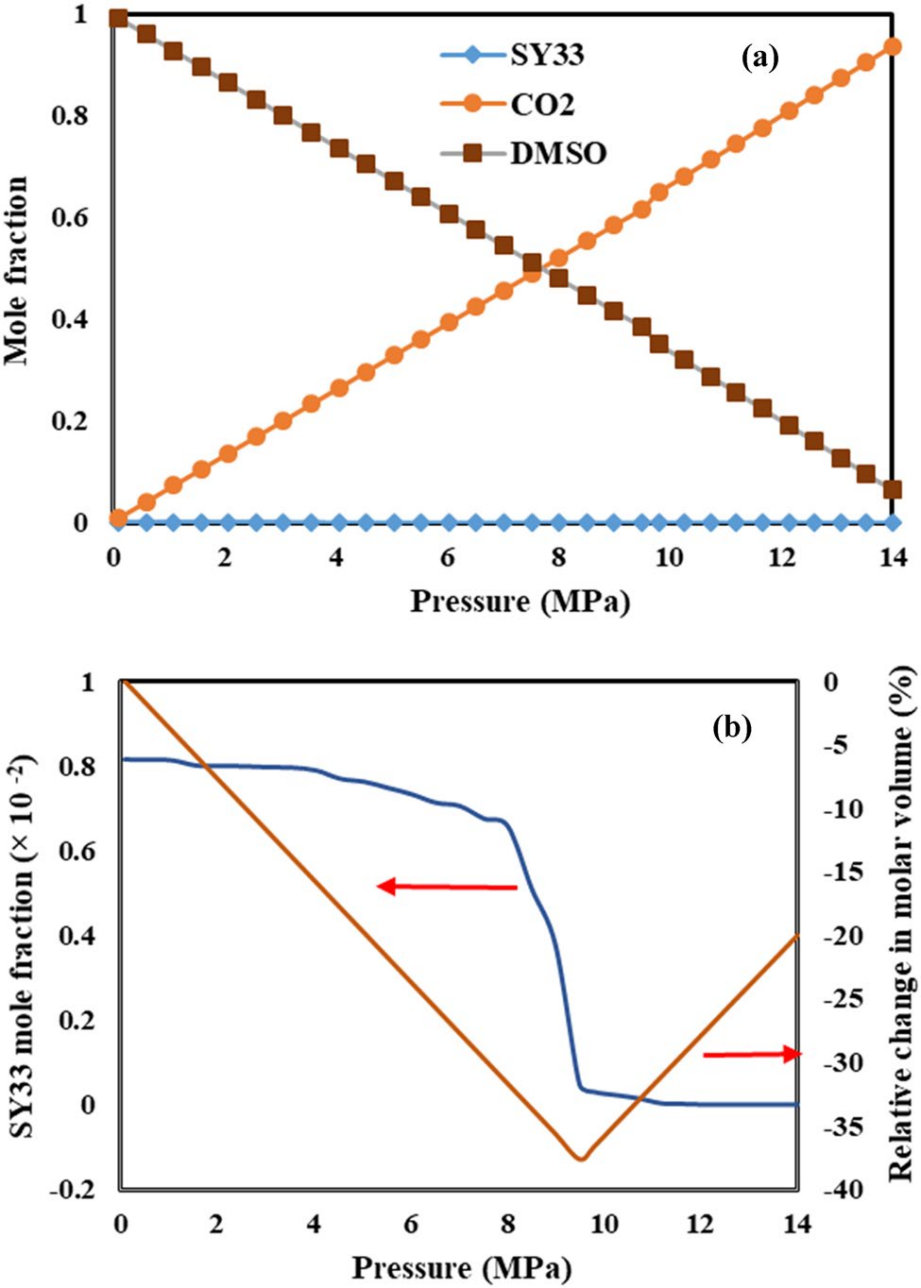
Mole fraction of components in the liquid phase of the ternary system of CO_2_–DMSO–SY33 at 328 K, calculated by the PR-EoS, and **(b)** Mole fraction of SY33 along with relative change in molar volume at 328 K.

Variation the concentration (mole fraction) of antisolvent (CO_2_), solvent (DMSO) and both of solutes in the GAS process at 328 K are calculated by PR-EoS and the results are shown in Figs. 8 and 9 for AV3RN and SY33, respectively. As seen in these figures, trend of mole fractions variations in both of ternary systems are the same, where in increment CO_2_ mole fraction and decrement DMSO mole fraction with increasing pressure were observed, simultaneously. Also for detailed interpretation, variation the mole fraction of dissolved AV3RN and SY33 in DMSO solution along with the change of relative molar volume of this liquid phase at 328 K is shown in Figs. 8b and 9b, respectively. Solutes solubility (mole fraction) in liquid phase gradually reduces by initiation CO_2_ injection into DMSO solution. By further pressure enhancement, severe drop in solutes solubility is observed in the P_min_ of each system (9.8 MPa for AV3RN and 9.5 MPa for SY33). Consequently, solutes mole fraction decreases to about zero, which confirms the precipitation of almost all of the solutes. This clearly approves that operational pressure of GAS process for each ternary system should be selected higher than its related P_min_ value. It is worth noting that trend of variations the mole fractions of CO_2,_ DMSO and both of pigments in the liquid phase of both of ternary systems in other temperatures is the same of 328 K, which are not shown here.

Experimental studies of Dixon and Johnston^50^ on determination the mole fraction changes of CO_2_ as antisolvent, toluene as solvent and naphthalene and phenanthrene as solutes have also the same results of our research. Thermodynamic modelling with regular solution theory and expanded liquid equation of state models confirmed their experimental results, too. Peters et al*.*^36^ calculated the change of naphthalene and phenanthrene solutes of (CO_2_-toluene-naphthalene) and (CO_2_-toluene-phenanthrene) ternary systems with PR-EoS. They simultaneously presented the variation of relative molar volume change of the solution and solutes mole fraction vs. pressure in one chart. It is completely obvious that in pressures higher than reported P_min_, the solutes concentration approaches to zero. Variation the solubility of ampicillin as a function of pressure in a ternary system of CO_2_-DMSO- ampicillin was calculated via PR EoS, by Ghoreishi et al*.*^30^. A slight increase in ampicillin solubility (mole fraction) in the liquid phase in pressure increment to about 4.5 MPa, and after that, a sharp decrease of its solubility have observed in the pressure range of 4.5–7.3 MPa. The minimum ampicillin solubility was indicated at the pressure above 7.3 MPa, which is equal to calculated P_min_ value of this ternary system. Change of CO_2_, toluene and naphthalene in liquid phase of corresponding ternary system was experimentally measured and thermodynamically predicted (PR-EoS and SRK-EoS) by Pahlavanzadeh et al*.*^26^. Also, they did the same work for CO_2_, ethanol and acridine system. In both of systems, sharp decrement of solutes (naphthalene and acridine) and solvents (toluene and ethanol) solubility were observed at calculated P_min_ value of each system. Reduction of salicylic acid mole fraction in CO_2_—1-propanol—salicylic acid ternary system at 288 K was experimentally determined and thermodynamically predicted by Peters et al*.*^51^. They also reported the same trend for salicylic acid and benzoic acid mole fraction variation at 313 K in CO_2_—1-propanol—salicylic acid and CO_2_-acetone-benzoic acid ternary systems, respectively^52^.

## Conclusion

In current study, the Peng–Robinson equation of state (PR EoS) was utilized for determination the phase equilibrium of the binary (CO_2_-DMSO) and both of ternary (CO_2_-DMSO-anthraquinone Violet 3RN (AV3RN)) and (CO_2_-DMSO-solvent yellow 33 (SY33)) systems. The critical properties of AV3RN and SY33 were calcuated using group contribution methods. The optimal operational condition (pressure and temperature) for precipitaion of AV3RN and SY33 nanoparticles via the GAS process were specified by the plot of relative liquid phase molar volume variations (computed with the definition presented by de la Fuente Badilla et al*.*) vs. pressure at the operational temperature. The minimum pressure of the (CO_2_-DMSO- AV3RN) and (CO_2_-DMSO-SY33) ternary systems were determined 7.80, 8.57, 9.78, 10.46 MPa and 8, 8.63, 9.5 and 10.77 MPa at 308, 318, 328 and 338 K, respectively. As is evident, temperature increment causes to increasing the minimum pressure value. Finally, variations the mole fractions of CO_2_, DMSO, AV3RN and SY33 of each ternary system were determined by the PR-EoS. By increasing dissolved CO_2_ concentration in the DMSO solution, solutes solubility reduces to about zero, which confirms the precipitation of almost all of the solutes. Sharp decrement of solutes solubility at pressures higher than P_min_ value of each ternary system approves that operational pressure of GAS process should be selected above this pressure.

## Data Availability

We perform the numerical study of the GAS process with the supercomputers and validated the obtained results with the available experimental reports.

## List of symbols

*PR*: Peng-Robinson EoS
*α(T)*: Energy parameter of Peng Robinson EoS (Nm^4^ mol^−2^)
*b*: Volume parameter of Peng Robinson EoS (m^3^ mol^−^^1^)
*y*_*i*_: Mole fraction of component *i* in vapor phase
*x*_*i*_: Mole fraction of component *i* in liquid phase
*k*_*ij*_: Binary interaction parameters in the mixing rules
*l*_*ij*_: Binary interaction parameters in the mixing rules
*P*: Pressure (Pa)
*T*: Temperature (K)
*R*: Gas constant (Jmol^−1^ K^−1^)
*ν*: Molar volume of the phase (m^3^ mol^−1^)
*H*: Heat of fusion
P_min_: Minimum required pressure for precipitation in GAS process
AV3RN: Anthraquinone Violet 3RN
SY33: Solvent Yellow 33

## Greek symbols

*α(T*_*r*_: *ω),* Temperature-dependent function for the considered parameter of Peng Robinson EoS
*φ*: Fugacity coefficient
*ω*: Acentric factor
Δ: Property change

## Subscripts

*1*: Antisolvent
*2*: Solvent
*3*: Solute
*0*: Reference pressure
*c*: Critical property
*i*: Species *i*
*l*: Liquid
*v*: Vapor
*s*: Solid
*tp*: Triple point
